# Automated Classification of Circulating Tumor Cells and the Impact of Interobsever Variability on Classifier Training and Performance

**DOI:** 10.1155/2015/573165

**Published:** 2015-10-04

**Authors:** Carl-Magnus Svensson, Ron Hübler, Marc Thilo Figge

**Affiliations:** ^1^Applied Systems Biology, Leibniz Institute for Natural Product Research and Infection Biology–Hans-Knöll-Institute (HKI), Beutenbergstraße 11a, 07745 Jena, Germany; ^2^Friedrich Schiller University Jena, Fürstengraben 1, 07743 Jena, Germany

## Abstract

Application of personalized medicine requires integration of different data to determine each patient's unique clinical constitution. The automated analysis of medical data is a growing field where different machine learning techniques are used to minimize the time-consuming task of manual analysis. The evaluation, and often training, of automated classifiers requires manually labelled data as ground truth. In many cases such labelling is not perfect, either because of the data being ambiguous even for a trained expert or because of mistakes. Here we investigated the interobserver variability of image data comprising fluorescently stained circulating tumor cells and its effect on the performance of two automated classifiers, a random forest and a support vector machine. We found that uncertainty in annotation between observers limited the performance of the automated classifiers, especially when it was included in the test set on which classifier performance was measured. The random forest classifier turned out to be resilient to uncertainty in the training data while the support vector machine's performance is highly dependent on the amount of uncertainty in the training data. We finally introduced the consensus data set as a possible solution for evaluation of automated classifiers that minimizes the penalty of interobserver variability.

## 1. Introduction

The identification and enumeration of circulating tumor cells is an important tool for evaluation of the disease progression in especially breast cancer [1–3] and is also under consideration as a diagnostic tool in various other types including lung and colorectal cancer [4–6]. The type of CTCs found also serves as a potential marker for changes in the chemotherapy resistance of a cancer [7]. The extreme rarity of CTCs in patient blood, typically one CTC per 10^8^ blood cells [8], makes both collection and detection of these cells extremely challenging. The collection of CTCs from peripheral blood is in a majority of studies done by antiepithelial-cell-adhesion-molecule (EpCAM) antibody-coated isolation systems [5, 9], but also other types of immunomagnetic devices [10, 11], density gradient centrifugation [12], and membrane filtration [13] are used for CTC enrichment. The detection of CTCs after collection is done by immunocytological staining or polymerase chain reaction (PCR) [14]. In the case of immunocytological staining the standard method of CTC enumeration is manual counting either at the microscope or from microscopy images [15, 16]. However, progress was lately made in using machine learning techniques for the detection of CTCs from fluorescence microscopy images [17, 18]. In these studies, as well as in any study applying classifiers to data, manual labelling was used for validation and also for training using (semi)supervised training regiments. The use of computational methods, in this case machine vision, makes the screening of the vast amounts of data that is readily available today quicker and more efficient. Instead of having a highly trained expert performing the time-consuming task of looking at numerous images, this can be done by the computer. Even if the computer is not able to completely take over the manual analysis it can at least screen the image data for regions of interest and provide a second opinion in difficult cases.

This paper builds on the previous result in enumerating CTCs in images using image analysis techniques combined with support vector machines (SVMs) and naïve Bayesian classifiers (NBCs) [18]. Data for the study was collected with a* functionalized and structured medical wire* (FSMW) [19] that is a CE-certified medical device for the isolation of CTCs. Human carcinoma cells expresses the epithelial cell adhesion molecule (EpCAM) on their surface while this molecule is absent from the surface of haematological cells [20–22]. The FSMW is functionalized with anti-EpCAM antibodies and was inserted into the cubital vein of a patient through a standard 20 G intravenous cannula, where it was left for 30 minutes collecting CTCs from the blood that flows past [19]. After cell collection the FSMW was fluorescently stained and microscopy images were made in which we aim to enumerate CTCs. Ideally only CTCs should adhere to the FSMW but because of the many blood cells compared to CTCs, even the unlikely event of catching a blood cell occurs regularly. The first step in the analysis was to identify regions of interest (ROIs) which are candidates as CTCs but may in fact also be a blood cell, some kind of debris or a staining artifact. In the previous study we concluded that both SVMs and NBCs achieved an accuracy of CTC detection in the range of 85–90% after ROIs were identified [18]. In that study, the annotation used for evaluation of classifier performance and training of the classifiers were based on the manual classification of the ROIs by one author (CMS).

The use of different machine learning and machine vision techniques is an active research field with the aim of making disease diagnosis more accurate and efficient [23]. Especially in the diagnosis and treatment evaluation of different cancer types, including but not limited to prostate [24, 25] and colorectal cancer [26], automated algorithms are used. However, interobserver variability is a known issue in diagnostics of different cancer types and a disagreement of more than 15% is not uncommon when multiple observers, normally all trained experts, are interpreting patient image data of different types [27–29]. When training and evaluating an automated classifier the labels provided by observers are of great importance as any inconsistencies will affect the performance of the classifier. In this study, we investigated how uncertainty in annotation, so called label noise, affects the performance of automated classification using a random forest (RF) and a SVM and relate that to the performance of earlier studies [17, 18]. Interobserver variability for disease progression using CTCs is reported to be as low as 1% but is then related to the question if the patient has more than 4 CTCs per 7.5 mL blood [27]. When considering the manual classification of images of possible CTCs, Scholtens et al. presented that observers disagree on approximately 15% of the data points [17]. To investigate how this variability affects the estimated performance of the classifiers, we in this study carefully identified possible label noise through analysis of the manual annotation. Moreover, a consensus annotation was identified and training and testing of the classifiers with a controlled amount of label noise in both training and test sets was evaluated.

## 2. Materials and Methods

### 2.1. Image Data

**Figure 1:**
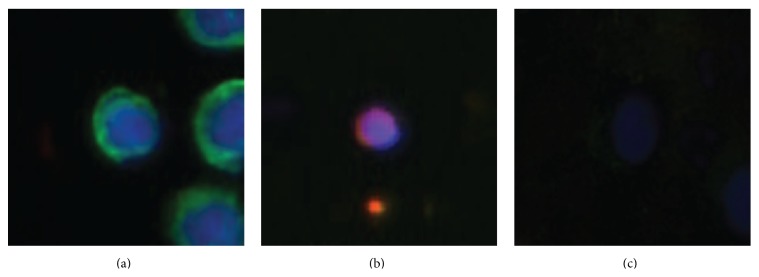
Examples of image cutouts classified with regard to the most central object being a CTC or not. (a) All eleven human observers agree that this is a CTC. (b) All eleven observers agree that this is not a CTC. (c) Six observers classify that this is a CTC while the other five say that it is not a CTC.

The data set used for this study was the same as used in our earlier publication [18], where CTCs were captured using the FSMW both* in vivo* and* in vitro* [19]. After collection the FSMW was fluorescently stained for cell nuclei (blue), EpCAM, or cytokeratins (green) and counterstained for CD45 (red) in order to differentiate between CTCs and blood cells that may have attached to the wire. Images were taken using a 10x ocular and 10x, 20x, or 40x objective resulting in 1.0 *μ*m^2^, 0.5 *μ*m^2^, or 0.25 *μ*m^2^ pixel resolution of the images. CTCs are those cells that exhibit nuclear dye (blue) colocalized with the antibodies against cytokeratin and/or EpCAM (both green); see Figure 1(a). ROIs, for example, objects that may be CTCs and most likely at least some type of cell, were identified based on the blue signal that indicates positive staining of a cell nucleus. For full details of the collection, staining, imaging, and ROI identification we refer the reader to earlier publications using this data set [18, 19]. The data points used for CTC classification were obtained by cutting out an image with area 100 × 100 pixels around the center of each identified ROI resulting in 617 data points from 61 original microscopy images.

### 2.2. Manual Annotation

Manual annotation was needed for both training and evaluation of the classifiers as well as for the determination of interobserver variability. The observers were instructed to determine if the most central object in each image cutout was a CTC or not. For an example of multiple objects occurring in the same cutout see Figure 1(a). According to guidelines used in earlier studies [18, 19], observers were instructed to count the object as a CTC if the nuclei (blue staining) were intact and the object showed positive staining for EpCAM or cytokeratin (green staining). The blue and green staining had to be distinguishable from each other; for example, the nuclei and the EpCAM staining should be structured. While it was required that the nuclei should be intact, it was allowed for CTCs to have irregular shapes or be clustered. Any object that showed positive CD45 staining (red) was not to be counted as a CTC; see Figure 1(b). All *N*_obs_ = 11 observers (with 5/6 male/female) had normal or corrected to normal eyesight and no one had any known issues with color vision. Cutouts were presented on individual laptops in one session to avoid different light conditions and without any time restrictions. The order of the data points was random and the observers were instructed not to confer.

### 2.3. Data Preprocessing and Automatic Classification

As the cutouts have been taken at different magnifications we first normalized the image matrix to cover a region of the size of 2500 *μ*m^2^ around the center of each image cutout. This means that the cutouts had 100 × 100, 71 × 71, or 50 × 50 pixels, depending on if they were from an image taken with a 40x, 20x, or 10x ocular. We then applied a Gauss convolution filter with standard deviation *σ* = 1 pixel to the cutouts to reduce the effects of high frequency noise. The classifiers we used, SVM and RF, both required inputs with fixed dimensions and therefore all cutouts were downsampled to 50 × 50 pixels using the raster package in R (https://cran.r-project.org/package=raster). The color space of the cutouts were red-green-blue (RGB) when read but for the classification we transformed them to hue-saturation-value (HSV) using the grDevices package (https://stat.ethz.ch/R-manual/). This was done as HSV has a natural division between color dimensions (H and S) and intensity (V), which is not present in RGB space. In the HSV space we dropped the V dimension as preliminary tests revealed that this factor is not decisive in the classification of cutouts containing a CTC or not. The image matrix was then vectorized so that each cutout is then represented by an array with 5000 entries with the hue and saturation values of the cutout. For the rest of this paper any reference to automated classification of a data point or cutout will mean that this vector containing the hue and saturation values of a cutout was presented to the classifier.

The automated classifiers were implemented in R using the h2o interface (https://cran.r-project.org/package=h2o) for the RF and the kernlab package (https://cran.r-project.org/package=kernlab) for the SVM. The RF was an implementation of the Breiman forest [30] consisting of 500 trees. The SVM with radial basis function (RBF) kernel [31] had the parameters *C* = 2 and *γ* = 0.005, where *C* is the soft margin penalty and *γ* the inverted radius of the RBF. Parameters, number of trees as well as *C* and *γ* for the SVM, were optimized to give the highest accuracy possible on a subset of the data.

To get the classifier responses to the data, all data sets, that is, both the entire set of 617 cutouts and subsets that will be described, were divided into randomized folds. Training of the classifier was then performed on a number of folds and testing was then done on one or more folds that were not used for training. This was done iteratively with new folds chosen for training and testing until all data points were classified. For each subset of data the number of folds and how they were used for training and testing are described in the text where appropriate.

## 3. Results

### 3.1. Interobserver Variability Reveals Differences in Bias and Large Degree of Uncertainty

In the *N* = 617 data points the observers found on average 300 CTCs with the median being 318, but the number varied largely as can be seen in Table 1. The lowest number of CTCs was found by the observer MTF with 221 CTCs and the largest number was 354 CTCs observed by ST. The largest interobserver distance in an ordered list was between MB (223) and MP (281) with 48 CTCs, while the second largest distance was 17 between JP (330) and CMS (347). The initial conclusion is therefore that two observers, MTF and MB, had a much more conservative opinion on what was to be considered a CTC than the other observers, thereby minimizing the risk for false positive CTC annotation. The rest of the observers have a range of detected CTCs that corresponds to approximately 10% of the total number of data points presented.

**Figure 2:**
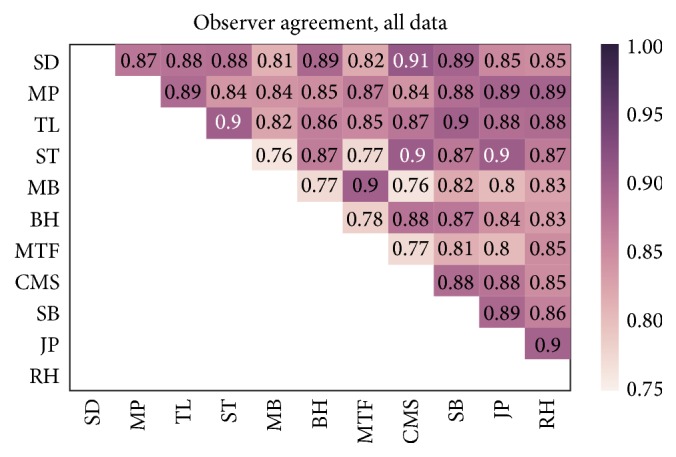
The agreement, *P*_Agr_, between observers across all *N* = 617 cutouts.

We define the agreement between observers *A* and *B* as

(1) $$\begin{array}{c} P_{\text{Agr}}\left(\;A,B\;\right)=1-\frac{1}{N}\overset{N}{\underset{i=1}{\sum }}\text{abs}\left(\;A_{i}-B_{i}\;\right), \end{array}$$

where *A*_*i*_, *B*_*i*_ ∈ [0,1] indicates the annotation of image cutout *i* as CTC (1) or no CTC (0) by the respective observer. If two observers agreed on all data points their agreement is one, whereas total disagreement gives the value zero. In Figure 2 a heat map showing the agreement between all observers is shown. It is worth noticing that the maximal agreement was 0.91 and that the average (median) agreement was 0.85 (0.87). This average agreement can be compared to the study by Scholtens et al. [17] that also had an interobserver agreement of 0.85, in that case across five observers. It should, however, be noted that the classification task in their study was not binary but objects were classified into one of five classes dividing the data into different types of CTCs and other objects including leukocytes. On the other hand, all observers in their study were referred to as experts, whereas in the present study the observers comprise experts as well as non-experts that were asked to identify CTC for the first time according to the criteria described in Section 2.

**Figure 3:**
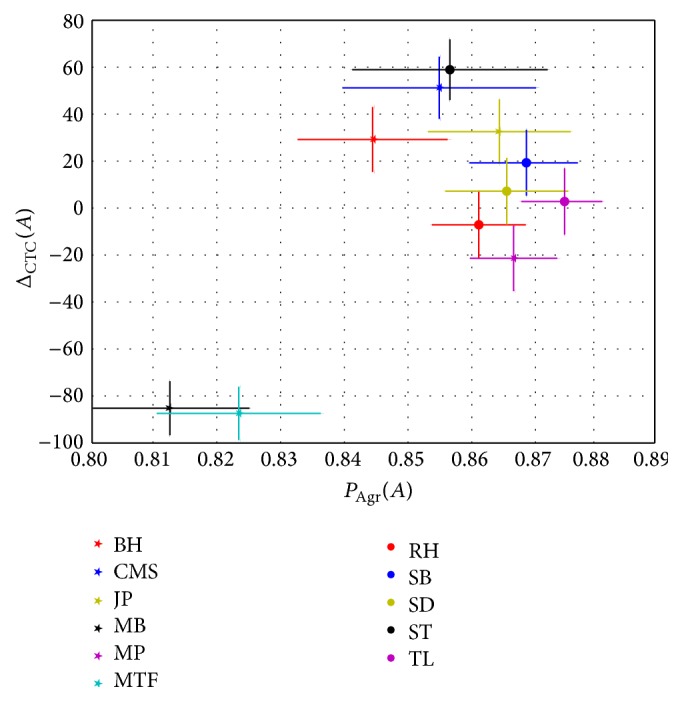
The average agreement for the observers plotted against the difference in number of found CTCs between the observers. Bars indicate the standard errors around the means.

In Figure 3 we present the average agreement per observer against the average difference in identified CTCs between one specific observer and all other observers. The average agreement between observer *A* and the others is defined as

(2) $$\begin{array}{c} P_{\text{Agr}}\left(\;A\;\right)=\frac{1}{N_{\text{obs}}-1}\underset{B\in \left(\text{observers}\neq A\right)}{\sum }P_{\text{Agr}}\left(\;A,B\;\right) \end{array}$$

and the mean difference in the number of CTCs found for observer *A* against all other observers is given by

(3) ΔCTCA=1Nobs−1∑B∈observers≠ANCTCA−NCTCB.

It can be seen from the clustering in Figure 3 that the two observers avoiding false positives in their indication of CTCs are isolated from the rest of the observers in both dimensions. Even though all participants were given identical instructions, both written and orally, these two observers made a different interpretation on how to annotate the data compared to the other nine observers. While the majority of observers tried to make a guess on cases where they were unsure, the observers MTF and MB always went for no CTC when unsure. For the two observers which avoided false positive CTC annotation the difference in the number of found CTCs seems to be the underlying reason for the low agreement with the other observers. The difference or similarity in number of identified CTCs however does not uniquely predict observer agreement. As an example we consider the two observers SD and TL, who have indicated 307 and 303 CTCs, respectively, that have an agreement of 0.88. On the other hand the observers MP and JP had an agreement of 0.89 although JP identified 49 more CTCs than MP. This emphasizes the need for a multidimensional and a multiobserver analysis regarding the interobserver agreement, rather than just looking at pairwise agreement and averages to identify observers with different biases.

The average agreement between any pair of observers was 85%; that is, *P*(*A* = *B*) = 0.85, and the assumption that the probability of agreement would be equal for each image cutout can be inserted into the Bernoulli distribution

(4) $$\begin{array}{c} P\left(\;A=B\;\right)=\left(\begin{array}{c} 2\\ 2 \end{array}\right)p^{2}=0.85, \end{array}$$

resulting in $p=\sqrt{0.85}\approx 0.92$. Based on this value, we estimate that all eleven observers should agree in 617 · *p* ^11^ ≈ 259 of the cases. In our dataset, all eleven observers agreed on 365 data points and we refer to these data points as the total consensus data set. This implies in turn an average pairwise agreement of *P*(*A* = *B*) = 0.91, which is significantly different (*p* < 10^−13^, Student's *t*-test) from the measured agreements in Figure 2. From these considerations we can draw the conclusion that the probability for disagreement is not the same for all image cutouts. To exemplify this, we in Figure 1 show a cutout for which all observers agreed of having a CTC (a) and one for which all agreed that there is no CTC (b). In the first case the conditions for a CTC are clearly fulfilled with the strong green staining and the clear integrity of the nucleus shown by the blue staining. The flanking objects were apparently not disturbing the observers. In Figure 1(b) the red staining identifies the object as a blood cell and all observers agreed that this is not a CTC. The third case, Figure 1(c), shows an example where the decision was split six versus five. The staining intensity in this cutout is lower than for the other cutouts and it is therefore hard to verify the integrity of the nucleus. Furthermore, it is difficult to determine if the green staining is structured enough for a positive CTC classification. It is also quite possible that some observers did not see the green staining at all due to the low color intensity.

### 3.2. Interobserver Agreement Does Not on Average Exceed 93% for Consensus Data

**Figure 4:**
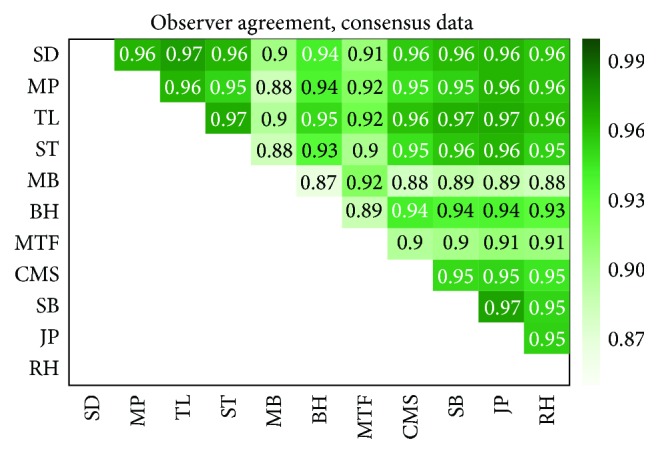
The agreement between observers for the 502 data points of the consensus data set.

The requirement that all observers should agree may be unnecessarily harsh as we may then discard data that a single observer made a mistake on. In studies where observers are not well supervised and possibly anonymous, as in the case of citizen science projects [32, 33], a single observer that misunderstands the task (or for some reason willingly gives false annotations) can severely damage the integrity of the data set. To determine how many observers we require to vote either CTC or no CTC, we defined a consensus limit, *c*, for which we say that consensus was reached. As the decision between CTC or no CTC is binary, we required that for the *N*_obs_ = 11 observers

(5) $$\begin{array}{c} P_{\text{bin}}=\overset{N_{\text{obs}}}{\underset{i=1}{\sum }}\left(\begin{array}{c} N_{\text{obs}}\\ c \end{array}\right){\left(\;\frac{1}{2}\;\right)}^{N_{\text{obs}}}<0.05; \end{array}$$

that is, the probability that *c* observers by chance annotated the cutout as containing a CTC or not should be less than 5%. In our case this means that *c* = 9 observers had to agree that the cutout does or does not contain a CTC for consensus to be reached and in our data set consensus was reached in 502 of the 617 cutouts (81%). For the consensus data set the interobserver agreement was naturally higher with mean (median) of 0.93 (0.95). In Figure 4 the agreement between observers for the consensus data set is shown as a heat map.

In the case of consensus data points, the observers that avoided false positive CTC annotation again had considerably lower number of CTCs than the other nine observers; see Table 1. Excluding the two observers with the no false positive bias (MB and MTF), the other nine observers are identified between 244 and 260 CTCs which gave a variation of around 3% of the total number of cutouts presented.

Given the distinctly different number of CTCs (see Table 1) identified by observers MTF and MB and their deviation from consensus (see Figure 4) the hypothesis that these two observers had a different bias than the others is further validated. However, instead of discarding the two observers as outliers, we decided that it may be rather interesting to see how annotations that arise from different biases affect the training of automated classifiers. In a setting where fewer observers are used it may not be possible to identify such differences in bias and it is also not sure that the differences in bias is restricted to a clear minority of observers.

### 3.3. Performance of Automated Classification Strongly Affected by Annotation Ambiguities

When evaluating automated classifiers different performance measures are used to show their agreement with an annotation considered to be ground truth. We have so far demonstrated that for certain data sets the annotation can vary strongly depending on the observer performing the annotation. The performance measures we use to evaluate the automated classifiers are defined with the help of correctly identified CTCs (TP), falsely identified CTCs (FP), objects correctly identified as not CTCs (TN), and CTCs that were not identified as such (FN). Our performance measures are then defined as accuracy Acc:

(6) $$\begin{array}{c} \text{Acc}=\frac{\text{TP}+\text{TN}}{\text{TP}+\text{FP}+\text{TN}+\text{FN}}, \end{array}$$

precision Pre:

(7) $$\begin{array}{c} \text{Pre}=\frac{\text{TP}}{\text{TP}+\text{FP}}, \end{array}$$

and recall Rec:

(8) $$\begin{array}{c} \text{Rec}=\frac{\text{TP}}{\text{TP}+\text{FN}}. \end{array}$$

Here, accuracy quantifies the fraction of correctly classified data points relative to all data points, whereas a high precision (recall) indicates a low number of falsely identified CTCs (missed CTCs).

In our earlier study [18], a support vector machine (SVM) achieved accuracy Acc = 0.89, precision Pre = 0.87, and recall Rec = 0.93 on the data set used here, given an annotation of data points performed by one observer (CMS). In the same study, a naïve Bayesian classifier (NBC) was trained without the use of labels, also known as unsupervised learning, which achieved accuracy Acc = 0.87, precision Pre = 0.85, and recall Rec = 0.92.

Our results from the interobserver variability study indicate that a different observer might have annotated the data quite differently. We divided the data set into five randomized folds, without any regard to whether the observers agreed on data points and train a random forest (RF) and a SVM on 3 of those and test on 1 fold. We got the average performance measures Acc = 0.86 ± 0.04, Pre = 0.83 ± 0.06, and Rec = 0.88 ± 0.08 across observers for the RF and the performance measures Acc = 0.86 ± 0.03, Pre = 0.85 ± 0.05, and Rec = 0.85 ± 0.08 for the SVM (see Table 2). Thus, the performance of the SVM and NBC in our previous study [18] was within one standard deviation of the numbers found here, for both the RF and the SVM. It should be noted that besides different implementations of the classifiers and the fact that only one annotation was used in [18], different features were also used. In our previous study, the features used were one-dimensional color histograms while in the present study the hue and saturation channels of HSV images were used. Taken together, we have used three automated classifiers (one RF, two SVM implementations, and one NBC) that performed almost equal on the data set. To add to this, the average interobserver variability was conspicuously close the accuracy of the classifiers, strongly suggesting that the performance of the classifiers was strongly influenced by annotation ambiguities.

To examine if and how differences in annotation affected the classifiers' performance, we split the data set into the total consensus data set that can be considered ground truth (GT) with 365 data points and a part with probabilistic annotation (probGT) containing the remaining 252 data points. From probGT different annotations can be generated by assigning the label for each data point from a randomly chosen observer. On average 81 data points will change label between two probabilistic annotations. For classifier evaluation, GT was in turn split into three folds and the probGT into two folds, giving in total five folds with approximately the same number of cutouts. To get prediction by the classifiers we trained on two folds and tested on a third fold. The test fold was always one of the GT folds as we were here trying to separate the effects of uncertain labels in the test set from uncertainty in training labels. Averages and standard deviations were obtained by 50 repetitions of the training and testing across the folds with new annotations drawn for probGT between each repetition. When training on only GT folds, which do not change any labels between repetitions, we repeated the procedure 10 times to check if any randomness originated in the training of the classifiers.

In Table 2 the performances of the classifiers are listed as we introduced different amounts of uncertainty in the training data. If training and testing were done only on the GT part of the data, the RF achieved performance measures Acc = 0.98 ± 0.00, Pre = 0.98 ± 0.00, and Rec = 0.98 ± 0.00, whereas the SVM achieved performance measures Acc = 0.96 ± 0.00, Pre = 0.95 ± 0.00, and Rec = 0.96 ± 0.00. The standard deviations were less than 1% confirming that both classifiers were stable between training runs and any deviations of this magnitude would originate from annotation changes in the probGT folds. The RF performances did vary in the order of 0.1%, which is due to the probabilistic build of the forest. Compared with the values achieved on the full data set this was a clear improvement when we tested and trained on noise-free data.

If we, instead of training only on GT, took one fold from GT and one from probGT and then tested on one GT fold the RF achieved performance measures Acc = 0.96 ± 0.01, Pre = 0.94 ± 0.02, and Rec = 0.98 ± 0.01 and the SVM achieved performance measures Acc = 0.92 ± 0.01, Pre = 0.88 ± 0.02, and Rec = 0.95 ± 0.01. This means that the label noise during training generally decreased the performance with a stronger performance reduction for the SVM than for the RF. The performances of both classifiers were still better than that recorded on the entire data set where testing was done against partly probabilistic annotation. Even when we trained the RF on the two probGT folds, which we know has a high degree of label noise and it can be assumed that the data in probGT is of a lower quality than in GT, its performance measures were Acc = 0.94 ± 0.02, Pre = 0.89 ± 0.05, and Rec = 0.97 ± 0.03. An example of what we refer to as low quality data is the low color intensity cutout shown in Figure 1(c). For this setting the performance of the SVM clearly dropped to Acc = 0.81 ± 0.04, Pre = 0.75 ± 0.08, and Rec = 0.84 ± 0.07.

This nicely illustrates that the RF is more robust when faced with label noise than many other classifiers, as was shown in the comparison between RFs and decision trees by Breiman [30]. While the SVM performed well in the pure GT case, its performance dropped more rapidly than the RF when uncertainty was introduced. When the training data contained at least 50% certain cases the SVM still performed better than it did on the entire data set, but when only probGT was used the SVM dropped to considerably lower levels. For the RF the performance level seen for the whole data set was mainly because the classifier is tested on unreliable annotation; that is, the training on unreliable labels did have an effect but that is fairly mild in comparison.

### 3.4. Consensus Data Provides a Base for Classifier Evaluation

To at least partly solve the issue of uncertain annotation affecting the performance of the automated classifiers we evaluated the classifiers against the consensus data set. As discussed earlier, it is reasonable that the consensus data set is defined as cutouts for which at least nine out of eleven observers agree with each other, because in this case the probability for random annotation of cutouts as containing a CTC or not is less than 5%. In the case of five observers it would be required that all five observers agree in order to satisfy this condition. Thus, the consensus limit varies with the number of observers. When training and evaluating the classifiers against the consensus data set we split the data set of 502 consensus data points into four folds, trained on three of them and tested on the fourth.

**Figure 5:**
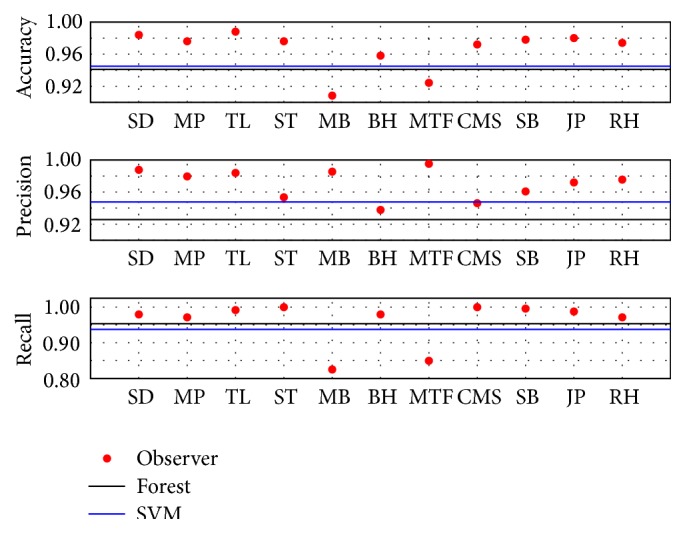
The accuracy, precision, and recall of observers (dots), RF (black line), and SVM (blue line) with the consensus annotation of the 502 cutouts for which consensus could be reached.

In Figure 5 the performances of the manual observers, RF and SVM versus the consensus labeling, are plotted. The performances of the RF and the SVM were close to each other. The SVM had a bit better precision, whereas the RF had a somewhat better recall. The performance measures for the RF were Acc = 0.94, Pre = 0.96, and Rec = 0.93, whereas the SVM had performance measures Acc = 0.94, Pre = 0.95, and Rec = 0.94. In comparison with our earlier study [18], we found an increase of the accuracy by approximately 5%, a precision increase by around 9%, while recall remained unchanged. Given the uncertainty in annotation that has been demonstrated in this study these values are much more representative performance measures for the task of automated classification of fluorescently stained CTCs. The majority of observers had better performances than the automated classifiers, but it should be noted that each observer had a vote when determining the consensus, whereas the RF and SVM did not. It should also be noted that none of the observers reached perfect performance in any of the measures. Hence, there exists not a subset of observers that could have served as a substitute for the consensus annotation.

In summary, the use of a consensus data set for training and evaluation of automated classifiers turned out to be a good option for evaluation of automated classification. In combination with the resilience of the RF to label noise in the training data, it seems that especially the test set has to be carefully chosen to give a correct evaluation of how well the classifier performs. The issue remains to find a good consensus data set as the manual annotation of data is hard to come by and time-consuming for the observers. This is especially the case when the annotation requires expert knowledge and experience in interpreting, for example, radiology images [28, 29].

## 4. Conclusions

As the use of computational methods is growing in cell biology, both for classification and modeling of biological systems [17, 18, 34, 35], we have in this paper investigated the effect of label noise caused by uncertain or faulty annotation on the performance of automated classification tasks. In total, eleven observers were asked to manually classify 617 image cutouts that may or may not contain CTCs. The rarity of CTCs in patient blood [7] can easily inflate the accuracy of any classifier due to the many true negatives (TN) that are normally present. The cutouts we used here were identified using a morphological classifier among approximately 35000 foreground objects found during initial image segmentation. The morphological classifier was designed for high recall so that very few CTCs were overlooked at the initial stage, for full details of the procedure see Svensson et al. [18]. For the 617 cutouts, it was revealed that observers agreed with probability 85% whether a CTC was present or not. This degree of agreement is comparable to the uncertainty often seen in manual assessment of medical image data [19, 23–29]. When only considering cutouts on which all observers agreed, the classifiers RF and SVM reached performance measures above 95% (see Table 2). This is considerably higher than the previously reported performances when attempting to automatically classify images of CTCs using RF and SVM [17, 18]. The RF turned out to be quite resilient to noise in the training data, even when using only uncertain data points in the course of training it performed better on the total consensus test set than classifiers in previous studies (see Table 2). The SVM was more sensitive to label noise in the training data and actually performed worse than it did when the whole data set was used for training and testing. These findings are in line with the findings of Breiman [30] that RFs are stable with regard to noise, although in that study RFs were only compared with decision trees. Going beyond that study, here we have demonstrated that they are also more stable than SVMs with a radial basis function (RBF) kernel. To test classifiers on data which is with a high probability incorrectly annotated or for which it cannot be uniquely decided on the actual class, as is the case for probGT, is of disadvantage for classifiers that cannot be corrected by machine learning algorithms. Any performance improvement above the uncertainty in annotation will be a type of overfitting and even if the achieved performance measures seem impressive the algorithm will most likely not perform well on other data sets. On the other hand, if the test data suffers from label noise it is of great importance to take this into consideration when evaluation any automated classifier.

Two very pressing questions remain to be investigated: (i) how to determine what is good data to use for training and testing the classifiers and (ii) how to detect and treat data that may occur in a clinical setting that is not appropriate for classification using the automated classification. Regarding the first question, we have shown that the creation of a consensus data set is a valid approach, but this normally requires a considerable effort from many observers to make the consensus statistically sound. In many cases these observer also have to be experts, for example, trained physicians that may not be very motivated to annotate data for machine learning algorithms rather than dealing with patients. It can be imagined that machine vision could step in to provide additional observers supplementing human observers. In this case care must be taken that the automated classification can be interpreted as an independent observer that is not getting slaved by human observers. This study suggests that RFs may be a strong candidate for this issue, because we have shown that noise in training data does not strongly affect the RF's performance on a total consensus test set. Another possibility is to use generative models which can be trained without labels [18] and which are therefore independent of the performance of the human observers. For the second question the ideal solution would be if the CTC imaging procedure would be (close to) perfect. In the case of CTC collection using FSMW, as done for the present data set, the cylindrical or spiral shape of the wire presents a considerable imaging challenge to get the entire surface in focus [18]. Even assuming a close-to-perfect data collection technique, it can be expected that clinical use will regularly produce data of a type that was not seen in training of the classifiers. A human observer could in such cases easily conclude that this is an uncertain case, whereas an SVM or RF will be forced to make a decision by design. In the machine learning literature there are methods for outlier detection and these may have to be implemented and developed to handle this task [36, 37]. For outlier detection to be efficient in this classification task, a further subgrouping of objects would probably be needed as the class representing objects that are not CTCs is a very inhomogeneous group of objects.

Instead of simply enumerating CTCs, as done here, it is desirable to determine subgroups within the CTC population, for example, to distinguish between apoptotic and viable CTCs [7, 17]. In order to do this, new sets of features may have to be identified that complement or even replace the color content of the cutouts. Examples of possible features would be further morphological quantities and Fourier-ring descriptors [38]. To apply machine learning to the subgrouping task would require more data than used here and a more rigorous manual classification performed by experts. As Scholtens et al. [17] demonstrated, we would in that case still be faced with a considerable interobserver variability that would require a handling along the lines presented in this study.

## Figures and Tables

**Table 1:** The number of CTCs identified in the data set by each observer.

Observer	SD	MP	TL	ST	MB	BH	MTF	CMS	SB	JP	RH
Number of found CTCs (*N*_CTC_), all data, *N* = 617	307	281	303	354	223	327	221	347	318	330	294
Number of found CTCs (*N*_CTC_), consensus, *N* = 502	244	244	248	258	206	257	210	260	255	250	245

**Table 2:** The performance of the classifiers when trained on two folds from probGT and GT in different combinations. It is cyclically tested on one GT fold that was not used for training.

	Training on two GT folds	Training on one GT fold and one probGT fold	Training on two probGT folds	Entire data set (across observers)
RF	Acc: 0.98 ± 0.00	Acc: 0.96 ± 0.01	Acc: 0.94 ± 0.02	Acc: 0.86 ± 0.04
	Pre: 0.98 ± 0.00	Pre: 0.94 ± 0.02	Pre: 0.89 ± 0.05	Pre: 0.83 ± 0.06
	Rec: 0.98 ± 0.00	Rec: 0.98 ± 0.01	Rec: 0.97 ± 0.03	Rec: 0.88 ± 0.08

SVM	Acc: 0.96 ± 0.00	Acc: 0.92 ± 0.01	Acc: 0.81 ± 0.04	Acc: 0.86 ± 0.03
	Pre: 0.95 ± 0.00	Pre: 0.88 ± 0.02	Pre: 0.75 ± 0.08	Pre: 0.85 ± 0.05
	Rec: 0.96 ± 0.00	Rec: 0.95 ± 0.01	Rec: 0.84 ± 0.07	Rec: 0.85 ± 0.08
